# Role of microRNAs in trophoblast invasion and spiral artery remodeling: Implications for preeclampsia

**DOI:** 10.3389/fcell.2022.995462

**Published:** 2022-10-03

**Authors:** Heyam Hayder, Yanan Shan, Yan Chen, Jacob Anderson O’Brien, Chun Peng

**Affiliations:** ^1^ Department of Biology, York University, Toronto, ON, Canada; ^2^ Centre for Research on Biomolecular Interactions, York University, Toronto, ON, Canada

**Keywords:** microRNA, trophoblasts, invasion, spiral artery remodeling, preeclampsia

## Abstract

It is now well-established that microRNAs (miRNAs) are important regulators of gene expression. The role of miRNAs in placental development and trophoblast function is constantly expanding. Trophoblast invasion and their ability to remodel uterine spiral arteries are essential for proper placental development and successful pregnancy outcome. Many miRNAs are reported to be dysregulated in pregnancy complications, especially preeclampsia and they exert various regulatory effects on trophoblasts. In this review, we provide a brief overview of miRNA biogenesis and their mechanism of action, as well as of trophoblasts differentiation, invasion and spiral artery remodeling. We then discuss the role of miRNAs in trophoblasts invasion and spiral artery remodeling, focusing on miRNAs that have been thoroughly investigated, especially using multiple model systems. We also discuss the potential role of miRNAs in the pathogenesis of preeclampsia.

## 1 Introduction

MicroRNAs (miRNAs) are involved in the regulation of gene expression and thereby exert a wide range of biological functions not only within the cells in which they are made but can also be secreted to mediate cell-cell communication (O'Brien et al., 2018). Many studies have shown that miRNAs are important regulators of placental development and trophoblast functions and their abnormal expression/secretion is associated with pregnancy-related disorders, including preeclampsia (PE) (Fu et al., 2013a; Farrokhnia et al., 2014; Mouillet et al., 2014; Zhang M. et al., 2016; Hayder et al., 2018; Skalis et al., 2019; Aplin et al., 2020).

The placenta is a transient organ that supports the development of mammalian embryos (Burton and Fowden, 2015; Maltepe and Fisher, 2015; Turco and Moffett, 2019). It regulates gas, nutrients, and waste exchange between the mother and the fetus, and secrets many pregnancy-associated hormones that are important for fetal growth and the progression of pregnancy (Iliodromiti et al., 2012; Camm et al., 2018). One of the key events during placental development is the invasion of trophoblasts into the decidua and subsequent remodeling of uterine spiral arteries to provide adequate placental perfusion to support the demands of the growing fetus (Lyall et al., 2013; Fisher, 2015). The remodeling of the spiral arteries by trophoblasts starts as early as 8 weeks of gestation (Burton et al., 2009; Whitley and Cartwright, 2010) and insufficient remodeling has been implicated in several pregnancy complications, including PE (Redman, 1991; Opichka et al., 2021; Pankiewicz et al., 2021; Staff et al., 2022). Therefore, knowledge of both spatial and temporal regulation of placental development is critical to our understanding of healthy pregnancy progression and the mechanisms underlying the development of many pregnancy complications and adverse fetal outcomes (Genbacev et al., 1997; Burton et al., 2007; Fisher, 2015; Hayder et al., 2018; Aplin et al., 2020).

Since their discovery, miRNAs have been shown to regulate all stages of normal pregnancy from implantation to labour (Renthal et al., 2013; Hayder et al., 2018). They are also reported to be dysregulated in many pregnancy-related diseases including PE (Fu et al., 2013a; Bidarimath et al., 2014; Mouillet et al., 2015; Munaut et al., 2016; Cai et al., 2017; Carreras-Badosa et al., 2017; Lykoudi et al., 2018; Ali et al., 2021). In particular, secreted miRNAs have been intensely studied as potential predictive and diagnostic markers for many of these conditions as well as possible therapeutic targets (Zou et al., 2018; Hornakova et al., 2020; Ali et al., 2021; Foley et al., 2021). As research into miRNA regulation and functions continues to evolve, our understanding of their role in placental pathophysiology advances.

In this review, we have provided an updated overview of miRNA biology and their role in regulating trophoblast invasion, spiral artery remodeling (SAR) and PE development, focusing on miRNAs that have been extensively investigated and/or those that have been studied using multiple experimental models.

## 2 Overview of microRNAs

### 2.1 Biogenesis

**FIGURE 1:**
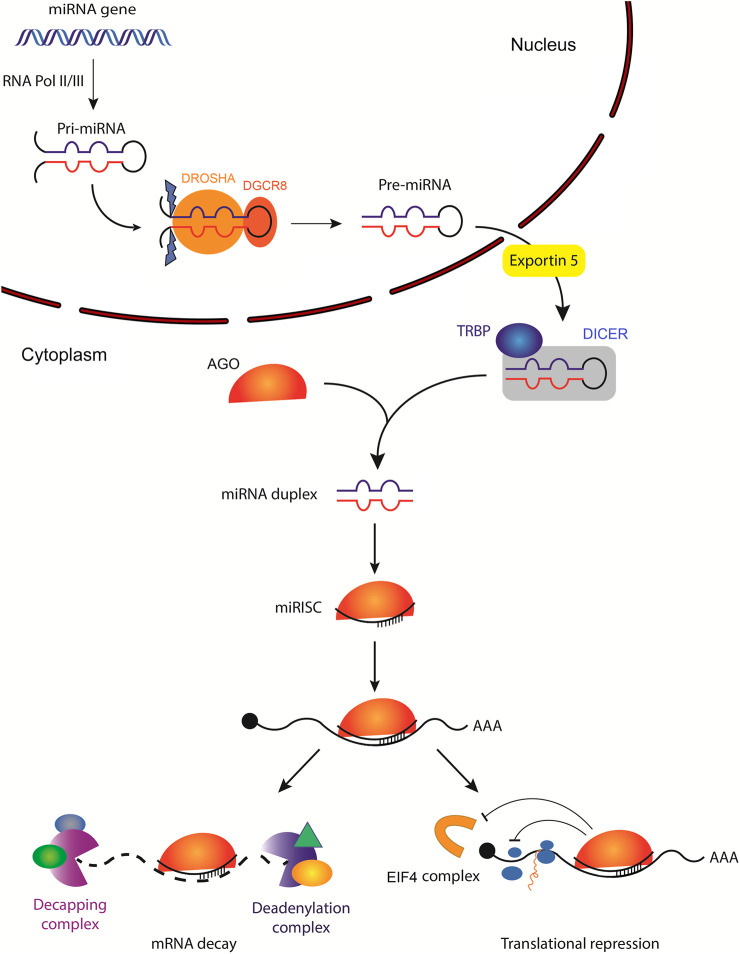
Biogenesis of microRNAs and their mechanisms of action. The primary miRNA (pri-miRNA) is transcribed by RNA polymerase II (less often RNA polymerase III) from a miRNA gene. Pri-miRNA is then processed by the DROSHA/DGCR8 complex to generate hairpin loop-containing precursor miRNA (pre-miRNA), which is exported from the nucleus by exportin-5. In the cytoplasm, pre-miRNA is first cleaved by DICER with the help of several RNA-binding proteins, including transactivation response element-binding protein (TRBP), which functions as a cofactor of DICER to ensure efficient processing of pre-miRNA. Subsequently, the mature miRNA duplex is unwound and one of the strands is loaded into the Argonaute (AGO) family of proteins to form a minimal miRNA-induced silencing complex (miRISC). Once miRISC is bound to its target mRNA, it can inhibit the initiation of translation by affecting the eukaryotic translation initiation factor 4 (EIF4) cap recognition and/or by inhibiting the formation of the 80S ribosomal complex. miRISC can also promote poly(A) deadenylation and mRNA decapping resulting in mRNA decay.

miRNAs are small non-coding single-stranded RNAs averaging 22 nucleotides in length (O'Brien et al., 2018). Canonically, a miRNA is transcribed by RNA polymerase II, or less often by RNA polymerase III, into a primary miRNA (pri-mRNA). This can either be from intragenic miRNA genes located mostly within the intron of a host gene, or from intergenic miRNA genes that exist independent of a host gene and are being regulated by their own promotor (Ha and Kim, 2014). Pri-miRNA stem-loop (hairpin) structure is then recognized by the microprocessor complex compromised of an RNA binding protein, DiGeorge syndrome Critical Region 8 (DGCR8) and a ribonuclease III enzyme, Drosha, which leads to the generation of precursor miRNA (pre-miRNA) (Denli et al., 2004; Han et al., 2004). Pre-miRNA is then exported into the cytoplasm by an exportin 5 (XPO5)/RanGTP complex and is further processed by Dicer, an RNase III endonuclease that removes pre-miRNA terminal loop forming a mature miRNA duplex (Zhang et al., 2004; Okada et al., 2009). Several RNA-binding proteins, including transactivation response element binding protein (TRBP), function as cofactors of Dicer to ensure accurate processing of pre-miRNA (Wilson et al., 2015; Fareh et al., 2016). The miRNA duplex unwinds into two single-stranded mature miRNAs, denoted by the -5p and -3p suffix, that can be loaded into the Argonaute (AGO) family of proteins (AGO1-4 in humans) in an ATP-dependent manner (Yoda et al., 2010; Medley et al., 2021) (Figure 1).

Although less common, non-canonical miRNA biogenesis is also observed (Abdelfattah et al., 2014). It can be classified into two groups, the Drosha/DGCR8-independent and the Dicer-independent pathways (Kim et al., 2016; O'Brien et al., 2018). These, for example, include miRNAs that originate from splicing the introns of messenger RNA (mRNA), mirtrons, or those that are processed from endogenous short hairpin RNA (shRNA), respectively (Ruby et al., 2007; Yang and Lai, 2011).

### 2.2 Mechanisms of action

Regardless of their biogenesis pathway, miRNAs regulate gene expression through the formation of the minimal miRNA-induced silencing complex (miRISC) which consists of a single-stranded mature miRNA guide strand and an AGO family protein (AGO1-4) (Kawamata and Tomari, 2010). The addition of effector complexes to minimal miRISC facilitates its function to negatively (and less frequently, positively) regulate gene expression at both transcriptional and posttranscriptional levels (Benhamed et al., 2012; O'Brien et al., 2018). The most well-studied mechanism of action is dependent on the interaction between miRISC and target mRNA at the 3′ untranslated region (UTR) (Valinezhad Orang et al., 2014; Jo et al., 2015; Salem et al., 2018; Wang N. Y. et al., 2019). This interaction between miRISC and target mRNA is typically stabilized by the 5′ seed region of the loaded miRNA (nucleotides 2–8) (Bartel, 2009; Ellwanger et al., 2011; Xu W. et al., 2014) and its association with a target sequence termed the miRNA response element (MRE) on the target mRNA (Behm-Ansmant et al., 2006; Nair et al., 2020).

An important early step in the regulation of target mRNA by miRISC is the formation of stable interactions between them (Chandradoss et al., 2015). This is dependent on miRISC and target RNA subcellular localization, RNA secondary structure, RNA-binding proteins, miRNA:RNA binding affinity, and miRNA:MRE copy number ratios. Within the cytoplasm, functional MRE are most commonly located within the 3′ UTR of mRNA (Huntzinger and Izaurralde, 2011; Ipsaro and Joshua-Tor, 2015) and to a much lesser extent the 5′ UTR (Zhang et al., 2018) and coding sequence (Fang and Rajewsky, 2011; Gu W. J. et al., 2013). miRNAs have also been detected in the nucleus to regulate gene transcription (Huang and Li, 2012; Liu H. et al., 2018). The affinity between miRNA and MRE itself significantly affects the stability of the interaction (Jo et al., 2015). Mismatches within the seed region, particularly within the first four nucleotides, substantially reduce miRISC interaction stability (Schirle et al., 2014; Chandradoss et al., 2015; Salomon et al., 2015), whereas supplementary interactions between MRE and miRNA within the central and 3’ regions have been shown, in some cases, to increase target specificity and stability (Sheu-Gruttadauria et al., 2019).

The mode of miRISC regulation is highly dependent on the set of proteins in complex with miRISC but in most cases, the formation of miRISC leads to the inhibition of gene expression via two major mechanisms: mRNA destabilization and translational repression (Figure 1). Following miRISC interaction with its cognate MRE within the cytoplasm, a miRISC adaptor protein, trinucleotide repeat-containing adaptor 6A (TNRC6A), interacts with poly(A) binding protein C (PABPC) (Liu et al., 2005; Huntzinger et al., 2013), poly(A)-nuclease deadenylation complex subunit 2 (PAN2/3), and the carbon catabolite repressor protein 4 (CCR4):NOT complex. Together, they localize the target mRNA poly(A) tail to miRISC, promoting efficient poly(A) deadenylation (Chen et al., 2009; Braun et al., 2011; Jonas and Izaurralde, 2015). After poly(A) deadenylation, the target mRNA is decapped by decapping protein 1/2 (DCP1/2) complexes, (Behm-Ansmant et al., 2006). mRNA decapping eventually leads to 5′ to 3’ exoribonuclease degradation by exoribonuclease 1 (XRN1) (Braun et al., 2012). Simultaneously, miRISC inhibits translation initiation by promoting the dissociation of eukaryotic translation initiation factor 4A1 (EIF4A1)/EIF4A2 and/or by inhibiting the formation of the 80S ribosomal complex (Behm-Ansmant et al., 2006; Fabian and Sonenberg, 2012; Meijer et al., 2013; Fukao et al., 2014). Although target mRNA degradation is typically observed following miRISC interaction, in some cases mRNA stability is left unchanged while inhibition of translation initiation results in decreased protein output (Pillai et al., 2005). Taken together, miRNA-bound AGO acts as a mediator of miRISC posttranscriptional regulatory potential in at least two primary ways: the miRNA-dependent localization of miRISC to target mRNA and the AGO-dependent recruitment of effector proteins that modulate target mRNA stability and protein output.

## 3 Overview of trophoblast differentiation, invasion, spiral artery remodeling, and preeclampsia

The main functions of the placenta are accomplished by trophoblasts, which are specialized cells of the placenta that play an essential role in embryo implantation and interaction with the decidualized maternal uterus (Imakawa and Nakagawa, 2017; Turco and Moffett, 2019). Abnormality in trophoblast differentiation and invasion, as well as spiral artery remodeling, is associated with various pregnancy-related disorders, such as PE.

### 3.1 Trophoblast differentiation

The human placenta originates from the trophectoderm, the outer layer of the pre-implantation embryo known as the blastocyst (Aplin, 2000). Following implantation, the trophectoderm undergoes proliferation and further differentiates into a branching network of villi that are in direct contact with the maternal circulation while maintaining a barrier between the fetal and maternal blood (Kaufmann et al., 2004; Wooding and Burton, 2008; Schmidt et al., 2015). The villous trees are made up of cytotrophoblasts (CTBs) covering the villous core, and a syncytiotrophoblast (STB) layer covering the villous surface that is in direct contact with the maternal blood. The CTBs are stem-like progenitor cells, which rapidly proliferate and fuse to give rise to the multinucleated STB layer. The STB layer of the placental villus functions as a physical barrier between the fetus and the mother to protect the fetus against vertical transmission of pathogens and immune attacks; it also regulates the exchange of O_2_, CO_2_, and nutrients between maternal and fetal blood, as well as secretes growth factors and hormones that are essential for normal pregnancy (Costa, 2016; Carrasco-Wong et al., 2021).

The CTBs also aggregate into cell columns at the tips of the anchoring villi. Cells at the distal region of the CTB column and in contact with the decidualized stroma lose most of their proliferative ability (Xu et al., 2002) and differentiate into invasive extravillous trophoblasts (EVTs). EVTs can be further grouped into interstitial EVTs (iEVTs) that invade the uterine stroma and endovascular EVTs (enEVTs) that invade spiral arteries to replace the endothelial cells (Pollheimer et al., 2018). Two possible origins of enEVTs have been proposed. First, they could be derived from the iEVTs surrounding the spiral arteries (Pijnenborg et al., 2006). Second, the shell CTBs that form the initial spiral artery plugs could differentiate into enEVTs which then start migrating within the spiral arteries in a retrograde manner (Pijnenborg et al., 2006; Burton et al., 2021). Some iEVTs can invade as far as the inner third of the myometrium, where they fuse to form multinucleated placental bed giant cells (Turco and Moffett, 2019). In addition, endoglandular EVTs (egEVTs) have also been identified as a potential subtype of iEVTs (Moser et al., 2010; Moser et al., 2015).

EVTs express a unique profile of adhesion molecules and histocompatibility antigens that facilitate their invasion ability as well as evasion of the maternal immune response. Villous CTBs anchored to the villous basement membrane retain their polarized epithelial state and express integrins ITGA6 and ITGB4 (α6β4) on their surface (Fisher and Damsky, 1993; Ji et al., 2013). As cells at the distal tip of the CTB column differentiate into EVTs, α6β4 is downregulated while integrin α5β1 is upregulated (Damsky et al., 1994). Integrin α1β1 is highly upregulated in EVTs that invade deeper into the decidua (Damsky et al., 1994; Damsky and Fisher, 1998). On the other hand, cadherin 1 (CDH1/E-cadherin) is downregulated in EVTs, resulting in the loss of cell-cell contact while invasive EVTs are characterized by the expression of cadherin 5 (CDH5/VE-cadherin) instead (Bulla et al., 2005; Ji et al., 2013). To avoid activating decidual natural killer (dNK) cells, iEVT express a distinct profile of HLA class I major histocompatibility complex (MHC) antigens that include HLA-C, HLA-E, and HLA-G (McMaster et al., 1995; Ferreira et al., 2017; Hackmon et al., 2017). HLA-E and HLA-G are thought to be vital for maternal tolerance of the semi-allogeneic fetus (Blaschitz et al., 2001). The placenta-specific HLA-G is highly expressed in invasive EVTs and can modulate the activity of not only dNK, but also macrophages and uterine T and B cells (Guillard et al., 2008; Ferreira et al., 2017).

### 3.2 Trophoblast invasion and spiral artery remodeling

During early pregnancy, EVT invasion is a crucial process for placental development, as the extent of the invasion is a key determinant for the quality of anchorage (Anin et al., 2004). This process is tightly regulated (Zhu et al., 2012) and insufficient invasion of EVTs is a hallmark of abnormal placentation and is associated with severe complications, such as PE, fetal growth restriction (FGR), and miscarriage. On the other hand, excessive invasion may result in placenta accreta and gestational trophoblastic disorders, including choriocarcinoma (Ning et al., 2019).

The precise mechanism underlying trophoblast invasion is not fully understood. Hormones, cytokines, growth factors, and proteinases are reported to be implicated in EVT invasion. Among them, matrix metalloproteinases (MMPs) play a critical role in mediating trophoblast invasion into the decidua by degrading the extracellular matrix (ECM) (Bischof et al., 2000; Anacker et al., 2011). For instance, MMP2 and MMP9 are two of the best-known key enzymes in trophoblast invasion (Huppertz et al., 1997; Staun-Ram et al., 2004; Jovanović et al., 2010).

Starting around 10 weeks of gestation, uterine spiral arteries undergo a remodeling process and are transformed from high-resistance vessels into dilated, low-resistance ones to ensure sufficient oxygen and nutrient supplies for the growing embryo (Anin et al., 2004; Lyall et al., 2013; Burton et al., 2021). This process involves coordinated actions of enEVTs, iEVTs, and decidual cells (Pijnenborg et al., 2006; Harris, 2010). The un-remodeled spiral artery contains endothelial cells surrounded by a layer of ECM and an outer layer of vascular smooth muscle cells (VSMCs). Early in the remodeling process, dNK cells and macrophages “prime” the vessels by disrupting the VSMCs and endothelial cell layers and triggering apoptosis (Smith et al., 2009). In the meantime, iEVTs invade the decidua into the areas surrounding the spiral arteries (Kam et al., 1999; Lyall et al., 2013) to remove VSMCs and replace the ECM with extracellular fibrinoid deposits, which are essential steps for normal spiral artery transformation (Kam et al., 1999; Harris, 2010; Lyall et al., 2013). iEVTs also release cytokines to help initiate the SAR process. For example, iEVTs release interleukin 6 (IL6) and C-X-C motif chemokine ligand 8 (CXCL8) to induce endothelial cells to secrete chemokines CCL14 and CXCL6 (Choudhury et al., 2017; Tao et al., 2019); in turn, the increased levels of CCL14 and CXCL6 recruit dNK cells and macrophages to further promote the remodeling process (Choudhury et al., 2017). Recent studies have also suggested that increased uterine arterial wall shear stress, as a result of pregnancy-associated increase in maternal blood volume and the decrease in vascular resistance, is one of the main physiological stimuli inducing uterine vascular remodeling (Ko et al., 2018; Khankin et al., 2021). The increase in wall sheer stress induces the remodeling, in part, by regulating endothelial cell signaling and the extent of arterial circumferential growth (Ko et al., 2018; Roux et al., 2020; Khankin et al., 2021).

The major function of enEVTs is to replace the endothelial cells lining the maternal uterine vasculature and this process is known as vascular mimicry (Ji et al., 2013). Interestingly, the invasion of enEVTs occurs in the arteries surrounded by perivascular iEVTs, suggesting that iEVT invasion could prepare for the subsequent enEVT invasion (Pijnenborg et al., 1983). Invasion of both iEVTs and enEVTs toward the spiral arteries is regulated in a spatiotemporal manner, as their invasive processes are terminated by 18–20 weeks of gestation and are limited to the inner third of myometrium (Pijnenborg et al., 1981). This remodeling process enhances placental perfusion in order to support the developing fetus.

### 3.3 Preeclampsia

According to the American College of Obstetricians and Gynecologists (ACOG) (2002) and the International Society for the Study of Hypertension in Pregnancy (ISSHP) (Davey and MacGillivray, 1988), PE is defined by *de novo* development of hypertension and multiple organ damage after 20 weeks of gestation (Lyall et al., 2013). This pregnancy-related disorder is a major cause of maternal and fetal morbidity and mortality and affects 2–8% of pregnancies worldwide (Huppertz, 2018; Phipps et al., 2019). The ISSHP groups PE into early-onset PE (EOPE), if patients are diagnosed and deliver before 34 weeks of gestation, and late-onset PE (LOPE, ≥ 34 weeks of gestation) (Raymond and Peterson, 2011; Tranquilli et al., 2013; Burton et al., 2019). More recently, detailed transcriptomic analyses of patients’ samples suggest PE can be subclassified based on molecular signatures; these subclasses can better describe the etiologies and the maternal–placental contributions to PE pathology (Leavey et al., 2016). Although the pathophysiology of PE is not completely understood, abnormal placental development is believed to play a central role in PE pathogenesis, particularly the EOPE (Noris et al., 2005; Brosens et al., 2011; Staff, 2019; Aplin et al., 2020).

PE is proposed as a two-stage disorder: the placental dysfunction stage and the maternal clinical syndrome stage (Redman, 1991; Staff, 2019; Carrasco-Wong et al., 2021). In the first stage, abnormal placental development due to defective trophoblast differentiation, shallow EVT invasion into the uterus and subsequent insufficient remodeling of spiral arteries leads to placental malperfusion. This in turn causes progressive oxidative stress of STB, characterized by erosion and damage of the surface of STB, increased shedding of inflammatory factors into the maternal circulation, decreased cell-cell fusion events, and reduced number of nuclei (Hubel, 1999; Cheng and Wang, 2009). Furthermore, some evidence suggests the existence of a switch from the normal apoptotic to the necrotic pathway making the shedded debris more pro-inflammatory compared to normal pregnancies (Paria et al., 2002).

**FIGURE 2:**
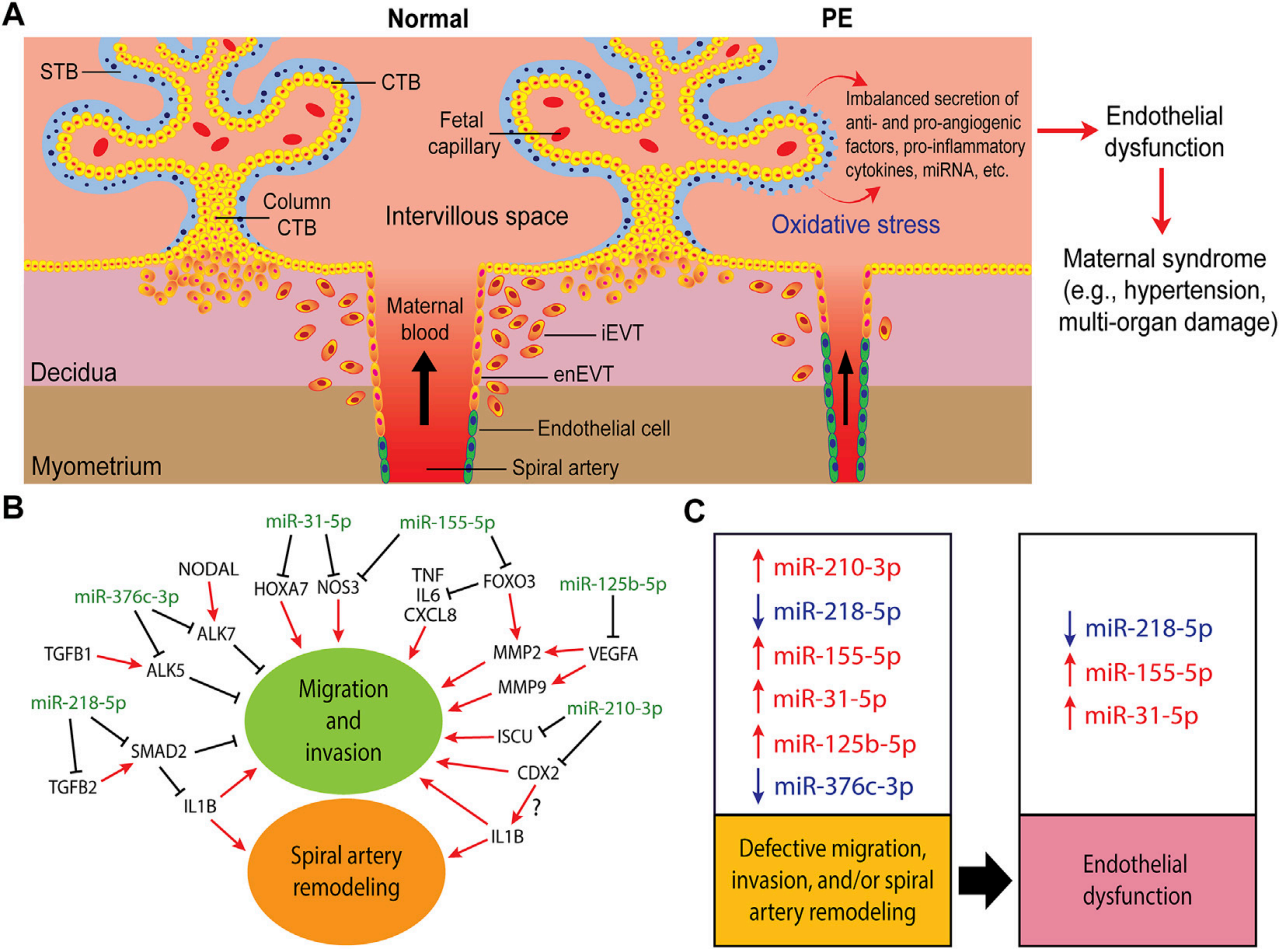
Trophoblast invasion, spiral artery remodeling, and their contribution to the pathogenesis of preeclampsia (PE). **(A)** In normal pregnancy, after implantation, the trophectoderm grows and differentiates into tree-like structures called villi which are the functional units of the placenta. A villous tree is made up of cytotrophoblasts (CTBs) covering the villous core, and a syncytiotrophoblast (STB) layer covering the villous surface that is in direct contact with the maternal blood. The CTBs also aggregate into cell columns at the tips of the anchoring villi. Cells at the distal region of the CTB column in contact with the decidualized uterine wall differentiate into invasive extravillous trophoblasts (EVTs). Interstitial EVTs (iEVTs) invade the decidua as far as the inner third of the myometrium. Some iEVTs move towards the maternal spiral arteries to help in their remodeling while endovascular trophoblasts (enEVTs) replace the endothelial cells lining these vessels. In PE, shallow EVT invasion and insufficient spiral artery remodeling lead to placental malperfusion and cause oxidative stress, resulting in the imbalanced secretion of pro- and anti-angiogenic factors, including miRNAs, into the maternal circulation. These factors lead to widespread endothelial dysfunction which results in the onset of maternal symptoms, such as hypertension, proteinuria, and/or multi-organ dysfunction. **(B)** A simplified regulatory network of some of the well-investigated miRNAs involved in regulating trophoblasts migration, invasion, and spiral artery remodeling. **(C)** The dysregulation of these miRNAs in PE (arrows denote up or down in PE) and their potential contribution into the proposed two-stages of PE pathogenesis: impaired placental development stage as a result of defective migration, invasion and spiral artery remodeling, and the maternal clinical syndrome stage due to endothelial dysfunction.

Placental malperfusion also leads to an imbalanced release of various pro-angiogenic, anti-angiogenic, pro-inflammatory, and anti-inflammatory factors into the maternal circulation (Benyo et al., 2001; Redman et al., 2014; Tannetta et al., 2017; Burton et al., 2019; Carrasco-Wong et al., 2021) (Figure 2A). This, in turn, provokes widespread endothelial dysfunction and results in the second stage of PE, the maternal clinical syndrome stage, characterized by the development of maternal hypertension, proteinuria, and/or multi-organ damage (Redman, 1991; Huppertz, 2018; Staff, 2019; Aplin et al., 2020; Carrasco-Wong et al., 2021). Soluble fms-like tyrosine kinase 1 (sFLT1), also known as soluble vascular endothelial growth factor receptor 1 (sVEGFR1), is commonly reported as being elevated in maternal serum in PE (Shibata et al., 2005). The upregulation of sFLT1 correlates with the downregulation of vascular endothelial growth factor A (VEGFA) and placental growth factor (PlGF) in PE, both of which are required for normal trophoblast function (Carty et al., 2008). In addition, decreased VEGFA levels contribute to the inhibition of EVT invasion and EVT-induced spiral artery remodeling (Norwitz, 2007). Studies of CTBs *in vivo* have also indicated that high level of sFLT1 decreases EVT invasiveness (Wang et al., 2009). The ratio of sFLT1/PlGF has been extensively evaluated as a biomarker for PE (Zeisler et al., 2016; Nikuei et al., 2020; Ohkuchi et al., 2021). Furthermore, many studies have also investigated the possibility of using miRNAs, secreted by trophoblasts via exosomes into the maternal circulation, as predictive markers of PE (Li H. et al., 2020; Matsubara et al., 2021; Palma et al., 2021; Xu et al., 2021).

Many studies have investigated changes in the placental transcriptome, including miRNA expression, in pregnancy-related disorders such as PE. Studying PE pregnancies that are also compromised by other pregnancy complications, such as small or large for gestational age babies, gestational diabetes, or FGR, can help to better understand the mechanisms that give rise to these complications and also identify unique molecular signatures as potential biomarkers and therapeutic targets for these disorders (Mayor-Lynn et al., 2011; Sõber et al., 2015; Luo et al., 2017; Zhong et al., 2019). Thus, the identification of reliable PE-specific biomarkers, including miRNA, is one of the main interests of PE research (Lala and Chakraborty, 2003; Carty et al., 2008; Rana et al., 2020; Stepan et al., 2020; Martinez-Fierro and Garza-Veloz, 2021).

## 4 microRNA regulation of trophoblast invasion, spiral artery remodeling, and preeclampsia development

To date, many miRNAs have been shown to regulate trophoblast invasion (Fu et al., 2013a; Doridot et al., 2013; Ji et al., 2013; Schjenken et al., 2016; Hayder et al., 2018), and cell-cell communication at the maternal-fetal interface (Morales-Prieto et al., 2020) and to be dysregulated in PE (Choi et al., 2013; Parada-Niño et al., 2022). Some miRNAs have also been implicated in SAR (Brkić et al., 2018; Hu and Zhang, 2019; Hayder et al., 2021; Pankiewicz et al., 2021). These studies range from the use of a single cell line, most commonly, HTR8/SVneo (Graham et al., 1993) to *ex vivo* and *in vivo* models with contradictory findings are often reported. As the HTR8/SVneo cells in different labs appear to be cultured under different conditions and gain different characteristics (Kilburn et al., 2000; Takao et al., 2011; Weber et al., 2013; Lee et al., 2016; Abou-Kheir et al., 2017; Brkić et al., 2020), it is important to use multiple experimental models when studying trophoblast behaviours.

When investigating the effects of miRNA regulation, there are multiple biological aspects that need be considered. A miRNA can target many genes; conversely, a gene can be regulated directly by many miRNAs. Also, there are numerous ways for miRNA to indirectly regulate genes. For example, a miRNA may target a set of genes, which in turn, regulate the expression of other genes. Therefore, to confirm the direct regulation of a target gene by a miRNA, the following studies should be performed. First, one should determine the direct interaction between the miRNA and the predicted MRE using a reporter assay (e.g., luciferase) and the effect of miRNA overexpression/inhibition on the mRNA and protein levels of the target gene. Second, the function of the target gene should be investigated. Finally, functional rescue experiments should be performed to determine if overexpression of the target gene will reverse the effect of the miRNA. Alternatively, or additionally, some researchers determine if silencing of the target gene can attenuate the effect of the miRNA inhibitor in their rescue experiments.

We searched PubMed for miRNAs that have been reported to regulate trophoblast invasion or SAR and are also dysregulated in PE. Table 1 lists miRNAs that have been reported to be either up- or downregulated in preeclamptic placentas or in exosomes isolated from these placentas and are found to regulate trophoblast migration, invasion, and/or SAR using at least 2 cell lines or 1 cell line with *ex vivo* or *in vivo* models. For target genes that have not been comprehensively investigated using expression analyses and functional rescue experiments, we include them in the table but denote them as partially validated. Table 1 is by no means an extensive list and we recommend some recent reviews on miRNAs in placental development and pregnancy complications (Lv et al., 2019; Skalis et al., 2019; Hemmatzadeh et al., 2020; Ali et al., 2021; Parada-Niño et al., 2022). Given the large number of papers published in these areas, we focus the discussion on the role of miRNAs that have been more extensively studied using multiple models in regulating trophoblast invasion and SAR (Figure 2B) and their potential contributions to the pathogenesis of PE (Figure 2C).

**TABLE 1:** Differentially expressed (DE) miRNAs in preeclampsia that affect trophoblast migration/invasion and/or spiral artery remodeling.

miRNA	DE in PE	Target genes	Regulation of invasion/SAR	Experimental model	References
miR-486-5p^##^	**↑**	*IGF1*	**↓** invasion, migration	HTR8/SVneo, TEV1	Salomon et al. (2017) Ma et al. (2021)
miR-135b-5p	**↓**	─	**↑** invasion, migration	HTR8/SVneo, HPT8	Vashukova et al. (2016) Zhao et al. (2021)
miR-24-3p	**↑**	FST*	**↓** invasion, migration	HTR8/SVneo, JEG3	Li et al. (2022a)
miR-3127-5p	**↑**	*HOXA7*	**↓** invasion, migration	HTR8/SVneo, JEG3	Li et al. (2022b)
miR-942-5p	**↓**	*THBS2*	**↑** invasion, migration	HTR8/SVneo, TEV1	Liu et al. (2022)
		*NLRP3*			Li et al. (2021a)
		*ENG*			Zhang et al. (2017)
miR-558	**↓**	*THBS2*	**↑** invasion, migration	HTR8/SVneo, JEG3	Li et al. (2022c)
miR-326	**↑**	*PAX8*	**↓** invasion, migration	HTR8/SVneo, JEG3	Zang et al. (2022)
miR-346	**↑**	*LRP6*	**↓** invasion, migration	B6Tert-1, HTR8/SVneo, JEG3	Zhang et al. (2020)
miR-19a-3p	**↓**	*IL1RAP*	**↑** invasion, migration	HTR8/SVneo, TEV1, *in vivo*	Wang et al. (2019a)
		lncPSG10P			
miR-378a-5p	**↓**	*NODAL*	**↑** invasion, migration	HTR8/SVneo, *ex vivo*	Luo et al. (2012)
miR-195-5p	**↓**	*FGF2*	**↑** invasion, migration	HTR8/SVneo, TEV1	Zhou et al. (2022a)
miR-128-3p	**↑**	*PCDH11X*	**↓** invasion, migration	HTR8/SVneo, JEG3	Wei et al. (2022)
miR-421	**↑**	*ZEB1*	**↓** invasion, migration	HTR8/SVneo, JEG3	Zhou et al. (2022b)
miR-218-5p	**↓**	*TGFB2*	**↑** invasion, migration, SAR	HTR8/SVneo, *ex vivo*,	Brkić et al. (2018)
		*SMAD2*	**↑** migration	HTR8/SVneo, Swan 71	Shan et al. (2022),
	**↑**	*LASP1*	**↓** invasion	HTR8/SVneo, JEG3	Fang et al. (2017)
miR-16-2-3p^##^	**↑**	*COL1A2*	**↓** invasion	HTR8/SVneo, primary trophoblasts	Tang et al. (2020)
miR-454-3p	**↓**	*EPHB4*	**↑** invasion, migration	HTR8/SVneo, primary trophoblasts	Wang and Yan, (2018)
miR-296-3p	**↑**	*CEMIP*	**↓** invasion, migration	HTR8/SVneo, JAR	Li et al. (2021b)
miR-126	**↑** (EOPE)	─	**↓** invasion, migration	HTR8/SVneo, JEG3	Liu et al. (2021a)
		lncLIN28A			Pan et al. (2021)
miR-149-5p	**↓**	*ERP44*	**↑** invasion, migration	HTR8/SVneo, JEG3	Liu et al. (2020b)
miR-183-5p	**↑**	*FOXP1*	**↓** invasion, migration	HTR8/SVneo, *in vivo*	Lai and Yu, (2020)
miR-138	**↑**	*RELA*	**↓** invasion	HTR8/SVneo, JEG3	Yin et al. (2021)
miR-142-3p	**↑**	*FOXM1*	**↓** migration	HTR8/SVneo, JEG3	Mao et al. (2021)
miR-95-5p	**↑**	*LRP6*	**↓** invasion, migration	HTR8/SVneo, B6Tert-1	Ni et al. (2021)
miR-15a-5p^##^	**↑**	*CDK1*	**↓** invasion, migration	HTR8/SVneo, *in vivo*	Wang et al. (2020b)
miR-181a-5p	**↑**	*IGF2BP2*─	**↓** invasion, migration	HTR8/SVneo, JAR	Huang et al. (2019) Wu et al. (2018)
miR-135a-5p	**↓**	*BTRC*	**↑** invasion, migration	HTR8/SVneo, TEV1	Wu et al. (2020a)
miR-133b^##^	**↓**	SGK1*	**↑** invasion, migration	HTR8/SVneo, HPT8	Wang et al. (2020a)
miR-106a	**↓**	─	**↑** invasion, migration	HTR8/SVneo, JEG3	Zhao et al. (2020)
miR-196a-5p	**↓**	lncSNX16	**↑** invasion, migration	HTR8/SVneo, TEV1, *in vivo*	Mi et al. (2020)
miR-384	**↑**	*PTBP3*	**↓** migration	HTR8/SVneo, JEG3	Zhou et al. (2020)
miR-101^##^	**↓**	*BRD4*	**↑** migration	HTR8/SVneo, *in vivo*	Cui et al. (2020)
miR-150-5p	**↑**	─	**↓** invasion, migration	HTR8/SVneo, JEG3	Zeng et al. (2020)
miR-139-5p^##^	**↓** (sPE)	*sFLT1* *PTEN*	**↑** invasion, migration	sPE primary trophoblastsHTR8/SVneo, *in vivo*	Huang et al. (2020) Liu et al. (2020a)
miR-206	**↑**	*IGF1*	**↓** invasion, migration	HTR8/SVneo, JEG3	Wu et al. (2020b)
miR-141-3p^##^	**↑**	─	**↓** invasion	HTR8/SVneo, JEG3	Ospina-Prieto et al. (2016)
miR-7-5p	**↑**	─	**↓** invasion, migration	HTR8/SVneo, 3A-sub E	Shih et al. (2019)
miR-10b-3p	**↓**	*LITAF*	**↑** invasion, migration	HTR8/SVneo, *in vivo*	Li et al. (2019)
miR-34a-5p	**↑**	MYC, NOTCH1, 2, 3*	**↓** invasion, migration	JEG3, PE primary trophoblasts	Sun et al. (2015) Liu et al. (2019)
miR-31-5p^##^	**↑**	*NOS3*	**↓** invasion, migration, placental artery relaxation	HTR8/SVneo, *ex vivo, in vivo*	Kim et al. (2018)
miR-137-3p	**↑**	*ESRRA*	**↓** invasion, migration	Primary trophoblasts	Lu et al. (2017)
miR-204-5p	**↑**	*MMP9*	**↓** invasion	BeWo, JEG3	Yu et al. (2015)
miR-125b-5p	**↑** (sEOPE) (LOPE) **↓**	*VEGFA*	**↓** invasion, migration	HTR8/SVneo, JEG3	Yang et al. (2016); Simsek et al. (2022); Sun et al. (2022)
miR-155-5p	**↑**	*NOS3*	**↓** invasion, migration, vasorelaxation	HTR8/SVneo, *ex vivo*	Sun et al. (2012); Li et al. (2014); Kim et al. (2017)
		*FOXO3*	**↓** invasion, migration	HTR8/SVneo, JEG3*, in vivo*	Luo et al. (2022)
miR-210-3p	**↑**	*ISCU*	**↓** invasion, migration	Swan 71, BeWo	Lee et al. (2011a)
		*CDX2*		HTR8/SVneo, *ex vivo*	Hayder et al. (2021)
miR-376c-3p	**↓**	*ALK5*, *ALK7*	**↑** invasion, migration	HTR8/SVneo, *ex vivo*	Fu et al. (2013b)
miR-517a/b/c	**↑**	*─*	**↓** invasion	Primary EVTs	Anton et al. (2015)

##: microRNAs, detected in extracellular vesicles from endothelial cells, mesenchymal cells or trophoblasts.

* These target genes were only partially validated.

PE: preeclampsia; sPE: severe PE; EOPE: early onset PE, LOPE: late onset PE.

### 4.1 miR-210-3p

Perhaps one of the best-known miRNAs involved in trophoblast invasion through targeting of multiple genes is hsa-miR-210-3p. This master hypoxamir (hypoxia-regulated miRNAs) is directly regulated by the binding of the hypoxia-induced transcription factor 1 α (HIF1A) to its promoter region (Chan et al., 2012), and by another hypoxia-regulated transcription factor, nuclear factor κ B subunit 1 (NFKB1) (Zhang et al., 2012). Early placental development normally occurs under low oxygen conditions (2–3% O_2_); this is important for trophoblast proliferation and to help maintain the trophoblast stem cell population. It is also advantageous in order to reduce reactive oxygen species production, thus limiting the oxidative damage to the zygotic DNA and maintaining pluripotency of embryonic cells through the stabilization of HIF family members (Burton et al., 2021). In one of our studies, we found that miR-210-3p expression was highest in first trimester (weeks 5–12) and lowest in third trimester (week 26–40) placental samples from normal pregnancies (Hayder et al., 2021). This higher first trimester expression level is consistent with the notion that miR-210-3p may play a role in trophoblast adaptation to a hypoxic environment during the early stages of placental development (Zaccagnini et al., 2013; Krawczynski et al., 2016) when trophoblasts have reduced mitochondrial function and rely heavily on glycolysis for ATP production (Kolahi et al., 2017; Burton et al., 2021), processes of which miR-210-3p is involved in regulating (Chen Z. et al., 2010; Muralimanoharan et al., 2012).

Several studies have reported that overexpression of miR-210-3p in different trophoblast cell lines and first trimester placental explants decreases trophoblast invasion by targeting various genes. For example, miR-210-3p targets iron-sulfur cluster assembly enzyme (*ISCU*), a key protein involved in mitochondrial function, and its downregulation results in decreased trophoblasts invasion (Lee D. C. et al., 2011; Colleoni et al., 2013). We recently reported that caudal-related homeobox transcription factor 2 (*CDX2*), a key transcription factor in trophectoderm formation, is also a target of miR-210-3p. We showed that overexpressing miR-210-3p or downregulating CDX2 decreased trophoblast migration and invasion, as well as EVT outgrowth into the Matrigel, in first trimester placental explants (Hayder et al., 2021). However, the mechanisms by which ISCU and CDX2 promote trophoblast invasion remain to be investigated.

In primary EVTs, miR-210-3p overexpression decreases cell invasion by activating the ERK/MAPK pathway (Anton et al., 2013), which is known to be activated by both hypoxia and lipopolysaccharide (LPS) treatment used to induce PE-like conditions (Park et al., 2011). We also found that overexpression of miR-210-3p reduced the ability of the HTR8/SVneo cell line to form endothelial-like networks, and decreased the mRNA expression of cytokines, interleukin one beta (*IL1B*), *CXCL8* and *CXCL1* (Hayder et al., 2021); which are important in regulating immune cells recruitment to SAR sites. However, using miR-210-3p inhibitor did not improve the network formation ability or cytokine expression levels in trophoblasts (Hayder et al., 2021). These findings suggest that while miR-210-3p may be dispensable for SAR, its excessive production could contribute to the defective SAR observed in PE.

The *in vivo* function of mir-210 has been investigated in several animal models; however, findings varied among species and environmental conditions. One study showed that neither mir-210 knockout nor placenta-specific overexpression of miR-210-3p in mice displayed changes in fetal and placental weight or morphology when compared to controls (Krawczynski et al., 2016). In another study, however, when mir-210 knockout mice were subjected to maternal hypoxic stress (10.5% O_2_) mimicking living at high altitude, a decrease in fetal weight and impaired placental development with reduced spongiotrophoblast layer and labyrinth fetal blood vessels were observed (Bian et al., 2021). In contrast, a study on pregnant sheep living at high altitude showed increased miR-210-3p levels in the uterine arteries and impaired uterine arterial adaptation to pregnancy by increasing pressure-dependent vascular resistance of these arteries (Hu et al., 2017). Interestingly, in humans, pregnant women living at high altitude have an increased risk of developing PE and FGR due to a lack of normal vascular adjustments to pregnancy including a lack of change in uterine vascular resistance, but whether miR-210-3p is involved in this was not verified (Palmer et al., 1999; Keyes et al., 2003). Thus, more studies are needed to fully understand the role of miR-210-3p in regulating placental development and maternal adaptation to pregnancy, especially under maternal stress conditions.

Caution should be applied when considering findings obtained from animal models in which PE has been induced. Namely, PE is exclusively a primate disorder and thus needs to be induced surgically or pharmacologically in mice. Also, while there are many similarities in placental structures and developmental processes between rodents and humans, there are still significant differences such as the extent of trophoblast invasion and the fact that the SAR process starts later in rodents compare to humans (Frazier et al., 2020). Moreover, placental malperfusion seen in preeclamptic placentas results in some areas being hyperoxic while others relatively hypoxic rather than the placenta just being hypoxic (Staff, 2019; Burton et al., 2021). This cycle of hypoxia-reoxygenation in PE could lead to differences in cellular responses to oxidative stress (Mukherjee et al., 2021; Pi et al., 2021; Qiu et al., 2021). Regardless, these studies demonstrated the emerging complexities of the role of miR-210-3p in placental development and the need to better understand its function in pregnancy.

The upregulation of miR-210-3p in the placenta and serum of women with PE is well-established (Pineles et al., 2007; Zhu et al., 2009; Liu et al., 2012; Muralimanoharan et al., 2012; Anton et al., 2013; Hayder et al., 2021; Jairajpuri et al., 2021). Studies have also shown that miR-210-3p levels in the serum of women who developed PE later in gestation were higher during the second trimester than in women who did not develop PE (Xu P. et al., 2014; Munaut et al., 2016), suggesting the possibility of using miR-210-3p as a predictive biomarker for PE. However, whether miR-210 upregulation is a consequence or cause of PE is yet to be determined; it is possible that the high miR-210-3p levels in PE can restrict trophoblast invasion and SAR thereby further contributing to PE pathogenesis.

### 4.2 miR-218-5p

Hsa-miR-218-5p is a highly conserved miRNA in mammalian species. In humans, miR-218-5p is transcribed from mir-218–1 (4p15.31) and mir-218–2 (5q35.1), which are located within the introns of the *SLIT2* and *SLIT3*, respectively (Mott and Mohr, 2015). Two precursors, mir-218–1 and mir-218–2, produce the same mature miR-218-5p and a precursor-specific miR-218-3p, miR-218-1-3p and miR-218-2-3p. In HTR8/SVneo cells stably transfected with mir-218–1, miR-218-5p was strongly upregulated while no significant change in miR-218-1-3p level was detected (Brkić et al., 2018), suggesting that miR-218-5p is the dominant mature miR-218 form. While little is known about the role of SLIT3 in the placenta, SLIT2 and its receptor, roundabout guidance receptor 1 (ROBO1), have been suggested to regulate trophoblast proliferation and differentiation and their aberrant signaling may be associated with various pregnancy-related disorders (Li et al., 2015; Li et al., 2017; Tiensuu et al., 2019). Interestingly, ROBO1, which is a functional target of miR-218-5p (Liu Y. et al., 2021), is upregulated in preeclamptic placentas (Liao et al., 2012).

We have investigated the function of miR-218-5p in trophoblast invasion, differentiation and SAR using various models and experimental methods. In trophoblast cell lines, stable or transient overexpression of miR-218-5p promoted migration, invasion, and the expression of various genes related to enEVT differentiation and SAR, as well as the formation of endothelial-like networks; meanwhile, the inhibition of endogenous miR-218-5p had the opposite effects (Brkić et al., 2018; Shan et al., 2022). In addition, miR-218-5p promoted the outgrowth of EVTs in first trimester placental explants. Finally, using a placental explants-decidua co-culture model, we demonstrated that overexpressing miR-218-5p in the explants increased the depth of trophoblast invasion into the decidua, induced the recruitment of immune cells, and enhanced the loss of smooth muscle cells surrounding the spiral arteries (Brkić et al., 2018). These findings suggest that miR-218-5p promotes enEVT differentiation and accelerates SAR. Mechanistically, we identified transforming growth factor β 2 (*TGFB2*) (Brkić et al., 2018) and its downstream mediator, *SMAD2* (Shan et al., 2022) as direct targets of miR-218-5p. We also showed that TGFB2 and SMAD2 exert inhibitory effects on enEVT differentiation (Brkić et al., 2018; Shan et al., 2022). Interestingly, inhibition of this pathway induced the expression and secretion of IL1B (Shan et al., 2022). We and other groups have shown that physiological concentrations of IL1B promotes trophoblast migration and invasion, and enEVT differentiation (Prutsch et al., 2012; Shan et al., 2022). Thus, one of the mechanisms by which miR-218-5p induces enEVT differentiation and SAR is to suppress TGFB2/SMAD2 signaling and to induce IL1B.

Several studies have reported that miR-218-5p is downregulated in preeclamptic placentas. Our lab has observed that miR-218-5p levels were significantly lower in PE placentas between 35–39 weeks of gestation than in their gestational age-matched controls in two sets of clinical samples collected in China and Canada (Brkić et al., 2018). Similarly, two miRNA profiling studies have reported the downregulation of miR-218-5p in placentas from women with severe PE and delivered at term (Zhu et al., 2009; Xu P. et al., 2014). Moreover, a recent study showed that miR-218-5p was significantly decreased in a rat model of PE while magnesium sulfate, which has been recommended for the treatment of PE (WHO, 2011), increased miR-218-5p levels (Zheng et al., 2022). In contrast, two other studies reported the upregulation of miR-218-5p in the plasma of PE patients at gestational age 20–40 weeks (Akgor et al., 2021) and in preeclamptic placental tissues collected primarily from 34–40 weeks of pregnancies (Fang et al., 2017). The reasons for these inconsistent findings are not clear. However, differences in gestational stages, sample size, clinical characteristics of the study subjects, and cell populations within the placental biopsies used for the miRNA measurement may contribute to such discrepancy.

While *in vivo* study directly investigating the contribution of miR-218-5p to PE pathogenesis remains to be conducted, based on *in vitro* and *ex vivo* studies, it is possible that downregulation of miR-218-5p could impede trophoblast migration, invasion, enEVT differentiation, and SAR, thereby contributing to the first stage of PE development. We also found that transfection of miR-218-5p into first trimester placental explants increased the secretion of pro-invasive and pro-angiogenic cytokines but decreased the secretion of an anti-angiogenic factor, soluble endoglin (sENG) (Brkić et al., 2018), which plays important roles in the second stage of PE development (Perez-Roque et al., 2021; Margioula-Siarkou et al., 2022). These findings, together with the observation that miR-218-5p is downregulated in a rat PE model but induced by magnesium sulfate to reverse the hypertension and proteinuria in these preeclamptic rats, support the notion that insufficient production of miR-218-5p is also linked to the development of maternal symptoms.

### 4.3 miR-155-5p

Hsa-miR-155-5p is a known pro-inflammatory miRNA encoded by the host gene, *MIRHG155*, previously known as the B-cell Integration Cluster (*BIC*) gene. Several transcription factors binding sites have been identified in the promoter region of *MIRHG155*, including NFKB1, SMAD4, interferon-sensitive response element (ISRE), interferon regulatory factors (IRF) and HIF1A (Mahesh and Biswas, 2019). Overexpressing miR-155-5p in trophoblasts decreased their invasion and migration while inhibiting miR-155-5p resulted in the opposite phenotype (Zhang et al., 2010; Dai et al., 2012; Li et al., 2014; Luo et al., 2022). A recent study reported that the forkhead-box class O transcription factor 3 (*FOXO3*), is targeted by miR-155-5p. It also showed that FOXO3 promoted trophoblast migration and invasion by decreasing the levels of tumor necrosis factor (TNF), IL6, and CXCL8 and by increasing the levels of MMP2 (Luo et al., 2022). These findings suggest that miR-155-5p can negatively regulate trophoblast invasion. However, direct evidence of the negative role of miR-155-5p in human SAR remains to be investigated.

Nitric oxide (NO) is an important signaling molecule that acts as a vasodilator but has also been shown to regulate trophoblast motility (Costa and Biaggioni, 1998; Harris et al., 2008). NO is produced in endothelial cells by the action of nitric oxide synthase 3 (NOS3), formerly known as endothelial nitric oxide synthase, eNOS. Both NO bioavailability and NOS3 levels are downregulated in PE (Albrecht et al., 2003; Kim et al., 2006; Guerby et al., 2019). It has been shown that miR-155-5p targets *NOS3* to inhibit HTR8/SVneo invasion (Li et al., 2014; Kim et al., 2017). Moreover, treatment of human vascular endothelial cells (HUVECs) with exosomes isolated from preeclamptic pregnancies and from miR-155-5p overexpressing BeWo cells suppressed *NOS3* expression (Shen et al., 2018) and that miR-155-5p decreased endothelium-dependent vasorelaxation (Sun et al., 2012). Thus, it is possible that by targeting *NOS3*, miR-155-5p reduces NO production, thereby impeding vasodilation and increasing blood pressure (Cardillo et al., 2000). This notion is supported by *in vivo* findings from a pregnant hypertension rat model, in which inhibition of miR-155-5p improved in blood pressure levels (Liu D. F. et al., 2018).

TNF is a well-known pro-inflammatory cytokine implicated in PE (Chen L. M. et al., 2010; Bellos et al., 2018). The inflammatory response serves a vital biological function and is finely controlled by many transcription factors, including NFKB1, to protect against tissue damage. However, prolonged and persistent activation of NFKB1 leads to increased production of cytokines, such as TNF and the interleukin family (Kim et al., 2013; Cornelius, 2018). This increase in pro-inflammatory cytokines is associated with impaired trophoblast invasion, endothelial dysfunction, and is considered a risk factor for PE (Vaughan and Walsh, 2012; Kim et al., 2018). TNF treatment has been reported to upregulate miR-155-5p expression, which in turn mediates TNF-induced downregulation of NOS3 (Sun et al., 2012). Interestingly, treatment with aspirin can prevent NOS3 downregulation by blocking both TNF-mediated miR-155-5p biogenesis and NFKB1-mediated *MIR155HG* expression (Kim et al., 2017). Aspirin is known to reduce hypoxia-induced sFLT1 release in trophoblasts and endothelial cells and to promote trophoblast invasion (Lin et al., 2019; Su et al., 2019). Low doses of aspirin are currently used to reduce the incidence of hypertensive complications during pregnancy (Henderson et al., 2014; Khanabdali et al., 2018) and the onset of EOPE if given daily to high-risk women before 16 weeks of gestation (Roberge et al., 2018; Ducat et al., 2019; Rolnik et al., 2022). In addition, several studies have reported the upregulation of miR-155-5p in preeclamptic placental (Lasabová et al., 2015; Azizi et al., 2017; Wang et al., 2021) and in maternal serum samples (Yang et al., 2017; Shen et al., 2018; Kim et al., 2020). Moreover, there is a strong positive correlation between placental and serum miR-155-5p levels suggesting a placental origin of serum miR-155-5p (Yang et al., 2017). When measuring extracellular miRNAs in maternal serum samples collected between 17 and 28 weeks of gestation, and before the onset of PE symptoms, a study showed that miR-155-5p was one of the best predictive biomarkers of PE (Srinivasan et al., 2020).

Together, these findings strongly suggest that aberrant overexpression of miR-155-5p can contribute to both stages of PE pathogenesis. Regardless of whether elevated miR-155-5p level are an actual cause or a consequence of other abnormal processes, it can further promote placental dysfunction by inhibiting trophoblast invasion. Additionally, higher miR-155-5p levels can induce endothelial dysfunction. Future studies should also explore the possibility of targeting miR-155-5p as a new preventive or therapeutic strategy for PE.

### 4.4 miR-31-5p

Several interesting studies have investigated the role of hsa-miR-31-5p in placental development. One study reported a negative role of miR-31-5p in HTR8/SVneo cell migration and invasion via targeting homeobox protein A7 (*HOXA7*), a member of the HOX family that has been shown to promote migration, invasion, autophagy and proliferation and inhibits apoptosis in HTR8/SVneo (Dong et al., 2020). Activated autophagy is an important mechanism for EVT invasion and vascular remodeling. It is thought to provide an “emergency” nutrient and energy supply for invading EVTs (Saito and Nakashima, 2014), especially under stress conditions such as starvation or hypoxia. In HTR8/SVneo and HUVEC co-culture model of vascular remodeling, autophagy-deficient HTR8/SVneo cell lines failed to replace endothelial-like networks formed by HUVEC; suggesting that autophagy is important for HTR8/SVneo to acquire endothelial-like phenotype (Zhang Y. et al., 2016; Nakashima et al., 2020; Li Y. M. et al., 2021; Li Z. J. et al., 2022). Thus, in light of the role of HOXA7 in promoting autophagy in EVTs, it is postulated that miR-31-5p-mediated downregulation of HOXA7 could limit deep invasion of EVTs and contribute to some of the phenotypes observed in preeclamptic placentas.

Treatment of HUVECs and HTR8/SVneo cells with TNF led to the upregulation of miR-31-5p (Kim et al., 2020) and this effect was mediated by NFKB1 (Kim et al., 2018). Interestingly, transfection of HUVECs with miR-31-5p mimic or treatment of HUVECs with TNF resulted in a decreased invasion of HTR8/SVneo co-cultured with these HUVECs. Further analysis revealed that miR-31-5p decreases trophoblast invasion by targeting *NOS3* (Kim et al., 2018). In addition to *NOS3*, miR-31-5p also negatively regulates HUVECs and human mammary epithelial cells (HMECs) proliferation, migration, and network-like formation by targeting endothelin receptor type B (*ETBR*), a receptor of endothelin-1 (ET1) which upon activation, causes vasodilation (Mazzuca et al., 2014; Li W. et al., 2020). *ETBR* is downregulated in PE (Li W. et al., 2020) and hypertensive pregnancies (Mazzuca et al., 2014) and abnormal *ETBR* expression contributes to microvascular dysfunction in postpartum women who have developed PE (Stanhewicz et al., 2017). miR-31-5p is upregulated in PE placentas (Li W. et al., 2020) and in serum of women with PE (Kim et al., 2018; Kim et al., 2020). In an *ex vivo* cultured model of placental arterial vessels from healthy pregnant women, TNF treatment or miR-31-5p mimic induced endothelial dysfunction and inhibited vasorelaxation (Kim et al., 2018) which are thought to underpin the onset of the clinical syndrome stage of PE (Staff, 2019; Aplin et al., 2020). Hence, by inhibiting trophoblast invasion and inducing endothelial dysfunction, elevated miR-31-5p levels in PE could contribute to both stages of the disorder but this requires further investigation.

### 4.5 miR-125b-5p

Hsa-miR-125b-5p, previously known as miR-125b, is generated from two different precursors, mir-125b-1 and mir-125b-2. In normal pregnancy, miR-125b-5p is expressed at higher levels in the third trimester than in first trimester placentas (Gu Y. et al., 2013). Also, miR-125b-5p overexpression reduces trophoblast invasion and migration (Li Q. H. et al., 2020; Xueya et al., 2020; Tang et al., 2021; Sun et al., 2022). This is mediated, in part, by targeting *VEGFA* (Sun et al., 2022), a well-known pro-angiogenic factor and a regulator of trophoblast function and placental angiogenesis (Amirchaghmaghi et al., 2015; Fan et al., 2021). VEGFA is also known to activate ERK1/2 signaling leading to upregulation of MMP2 and MMP9 to promote trophoblast invasion (Su et al., 2017; Wang et al., 2017). Moreover, placenta-specific overexpression of miR-125b-5p in mice decreased placental and fetal weights and increased abortion rate. Further analysis in these mice revealed that the observed phenotypes were due to a significant reduction in branch density of placental vasculature and in trophoblast invasion into the decidua and the spiral arteries (Sun et al., 2022). These findings strongly support an inhibitory role for miR-125b-5p in trophoblast invasion, placental angiogenesis and SAR.

Studies of miR-125b-5p expression in pregnancy complications vary based on the type and onset of these disorders. In preeclamptic placentas, one study reported that miR-125b-5p is upregulated in severe EOPE placentas (Yang et al., 2016) while another one showed downregulated miR-125b-5p in LOPE (Simsek et al., 2022), and a third study reported no difference (Inno et al., 2021). However, miR-125b-5p was consistently shown to be upregulated in umbilical cord blood (Simsek et al., 2022) and in serum of women affected by EOPE (Yang et al., 2016; Tang et al., 2021; Simsek et al., 2022). Interestingly, miR-125b-5p was also shown to be elevated at 12 weeks of gestation in women who developed PE later in their pregnancies, suggesting that miR-125b-5p miRNA could be used as a predicative marker for PE (Licini et al., 2021). The detection of aberrant upregulation of miR-125b-5p in first trimester, together with the findings that placenta-specific overexpression of miR-125-5p in mice resulted in limited trophoblast invasion into the decidua and spiral artery, strongly supports the role of this miRNA in PE development, particularly EOPE, and its potential as a therapeutic target for preventive PE strategy.

### 4.6 Chromosome 14 miRNA cluster

Chromosome 14 miRNA cluster (C14MC) is conserved among placental mammals. In humans, this cluster contains 54 miRNA genes (Welten et al., 2014; Nayak et al., 2018) with 84 mature miRNAs identified so far (Smith et al., 2021). It is located on chromosome 14q32 at the imprinted DLK1-DIO3 domain (mouse distal chromosome 12 domain cluster) where it can be expressed from the maternally inherited chromosome 14 (Seitz et al., 2004; Glazov et al., 2008; Morales-Prieto et al., 2013; Welten et al., 2014). Although many C14MC members appear to have variable expression patterns in the first trimester, the majority of them are expressed at a higher level in the second trimester than in other gestational stages (Morales-Prieto et al., 2012; Gu Y. et al., 2013; Gonzalez et al., 2021; Inno et al., 2021; Smith et al., 2021). Also, many C14MC miRNAs are detected in both fetal and maternal plasma in normal pregnancy (Paquette et al., 2018). As expected, heterozygous mouse pups with maternal, but not paternal, deletion of this maternally-inherited miRNA cluster exhibited partially penetrant neonatal lethality due to defects in the maintenance of energy homeostasis (Labialle et al., 2014), suggesting an essential role of these miRNAs during pregnancy.

The role of specific members of the C14MC in trophoblasts function and placental development has been investigated. For example, our lab reported that miR-376c-3p inhibits transforming growth factor β 1 (TGFB1) and NODAL signaling by targeting their respective type I receptors, activin receptor-like kinase 5 (*ALK5*) and *ALK7*. This, in turn, significantly increases HTR8/SVneo invasion and EVT outgrowth of first trimester placental explants (Fu et al., 2013b). We and others have shown that TGFB1 and NODAL negatively regulate trophoblast invasion and EVT outgrowth of first trimester placental explants (Nadeem et al., 2011; Luo et al., 2012; Shi et al., 2019; Brkić et al., 2020). miR-376c-3p is also downregulated in both placental and maternal plasma samples from PE patients compared to those from normal pregnancies (Fu et al., 2013b; Sandrim et al., 2016). Thus, lower miR-376c-3p levels would possibly lead to insufficient trophoblast invasion and thereby contributes to the development of PE. However, *in vivo* studies are needed to confirm this possibility.

Another member of C14MC is miR-494-3p; it promotes migration and invasion of trophoblast cell lines by targeting high-temperature requirement A serine protease 1 (*HTRA1*) (Ou et al., 2020). HTRA1 is a secreted protein that inhibits trophoblast migration and invasion (Ajayi et al., 2008; Fantone et al., 2021). HTRA1 interacts with HTRA3 to antagonize and degrade another HTRA member, HTRA4 that is known to promote trophoblast invasion (Wang et al., 2012; Chen et al., 2014; Fantone et al., 2021). *HTRA1* is upregulated in EOPE (Ajayi et al., 2008; Liu C. H. et al., 2018), and surprisingly, so is miR-494-3p (Zhao et al., 2022). However, HtrA1-deficient mice display smaller placentas and exhibit impaired SAR and FGR (Hasan et al., 2015). Thus, identifying other targets of miR-494-3p in human trophoblasts, can shed the light on the precise mechanisms by which miR-494-3p regulate placental development and its implication in PE.

In preeclamptic placentas, some of C14MC miRNAs are upregulated, while others are downregulated (Timofeeva et al., 2018; Devor et al., 2020; Inno et al., 2021); however, a detailed analysis of all miRNAs in this cluster is yet to be done. Due to the lack of studies on the specific role of many C14MC members in trophoblast invasion, it is unclear how these cluster miRNAs may contribute to the development of PE. Therefore, more studies, especially *ex vivo* and *in vivo* ones, are needed to further determine how C14MC members regulate placental development, if and how they are dysregulated in PE, and how they may play a role in PE pathogenesis.

### 4.7 Chromosome 19 miRNA cluster

Chromosome 19 miRNA cluster (C19MC) is a primate-specific miRNA cluster that is expressed almost exclusively in the placenta (Xie et al., 2014). It is located on chromosome 19q13.41 and contains 46 miRNA genes that are processed from RNA polymerase II non-protein coding transcripts (Bortolin-Cavaille et al., 2009). The C19MC is maternally imprinted and expressed exclusively from the paternally inherited chromosome 19 (Noguer-Dance et al., 2010). In primary trophoblasts isolated from normal term placentas, 41 out of the 46 C19MC miRNA genes were expressed and they represented the majority of miRNAs expressed in these cells as well as in the exosomes secreted by them (Luo et al., 2009; Donker et al., 2012; Lee et al., 2016). Temporally, studies have shown that C19MC miRNAs exhibit a dynamic gestational expression profile (Gottlieb et al., 2021); however, there are inconsistencies among different reports. One study showed that 46 mature C19MC miRNAs were higher in third trimester villous trophoblasts compared to first trimester cells (Donker et al., 2012; Morales-Prieto et al., 2012), while another study showed that the expression of some C19MC members were higher in first trimester samples (ranged 6–10 weeks) than in second trimester ones (ranged 11–23 weeks) (Smith et al., 2021). Nevertheless, C19MC miRNAs still constituted 15% of total miRNA transcripts at term placentas (Smith et al., 2021).

Some C19MC members displayed strong expression in first trimester human villous CTBs. Within the CTB column, C19MC expression levels gradually decreased from proximal to distal cells as CTBs differentiated into EVTs (Xie et al., 2014; Mong et al., 2020). Consonantly, many C19MC miRNAs were detected in JEG3, JAR and BeWo choriocarcinoma trophoblast cell lines but not in the EVT-like HTR8/SVneo cell line (Donker et al., 2012). Interestingly, ectopic stable expression of the entire C19MC in HTR8/SVneo reduced cell migration (Xie et al., 2014). Gene ontology analysis using the C19MC overexpressing HTR8/SVneo showed a downregulation of many genes involved in cell motility and adhesion (Xie et al., 2014). These findings suggest that C19MC exerts a negative regulatory role in EVT differentiation and migration. However, the role of individual C19MC members has not been well established. Only few studies have investigated the role of specific C19MC members in trophoblast function and done so using only a single cell line (Anton et al., 2015; Ding et al., 2015; Zhang et al., 2021; Zheng et al., 2021).

Recent studies suggest that C19MC plays important roles in placenta development. In human embryonic stem (hES) cells, C19MC expression is important for the differentiation of hES into human trophoblast stem cells (hTS). Using CRISPR/Cas gene editing tools, the authors further demonstrate that C19MC is critical in hTS maintenance (Kobayashi et al., 2022). The exact mechanism behind C19MC stem cell maintenance is uncertain but it may involve the C19MC-induced upregulation of pluripotent factors octamer-binding transcription factor 4 (*OCT4*) and fibroblast growth factor 4 (*FGF4*) (Mong et al., 2020). In addition, transgenic mice expressing the human C19MC miRNAs had also been developed. The C19MC transgenic mice had larger placentas with enhanced trophoblast proliferation and altered trophoblast migration (Mouillet et al., 2020). Compared with the wild type mice, the C19MC transgenic mice express a higher level of *Tpbpb*, a marker of the junctional zone glycogen cells (similar to human EVTs) and *Mmp1a*, a gene involved in trophoblast migration and invasion (Soncin et al., 2015; Mouillet et al., 2020). Thus, these findings appear to be at odds with those from the *in vitro* studies using human trophoblast cell lines. Further studies are required to elucidate the role of this miRNA cluster in trophoblast migration, invasion, and SAR.

In PE, many members of the C19MC were shown to be dysregulated with the majority of them being upregulated (Hromadnikova et al., 2013; Miura et al., 2015; Inno et al., 2021). For example, miR-512-5p, miR-515-5p, miR-517a/b/c, miR-518a/e/f-5p, miR-519a/b/c/e-5p, miR-519days/c-3p, miR-520a/b/c-5p and miR-520a-3p were consistently upregulated in PE (Anton et al., 2015; Ding et al., 2015; Zhang M. et al., 2016; Inno et al., 2021) while miR-524-5p and miR-525-5p were consistently downregulated (Zhang et al., 2021; Zheng et al., 2021). Given the large number of miRNAs within the C19MC cluster, the differential regulation of at least some cluster members and their potentially varying function, it can be expected that individual miRNAs in this cluster have distinct expression profiles throughout gestation. Nevertheless, C19MC appears to be critical for placental development in at least two key areas: the maintenance and proliferation of hTS and their differentiation into EVT. However, following EVT differentiation, C19MC expression may be deleterious by decreasing migration and modulating adhesion potential. Thus, differential expression of C19MC members in PE requires a high spatial resolution, particularly between villous trophoblast populations and EVT populations. It may be that the upregulation of C19MC in PE is compensatory, attempting to increase CTB cell number and STB integrity via STB differentiation. If the PE environment impairs C19MC downregulation following EVT differentiation, this may further exacerbate insufficient EVT invasion and SAR. Consequently, it is important that future research on this cluster obtain detailed analysis of the expression of all C19MC mature miRNAs in PE to be able to determine their involvement in PE pathophysiology and further our understanding of cluster miRNA dynamics.

## 5 Concluding remarks

Placental development is highly regulated and involves a complex interplay between fetal and maternal cells to ensure a successful pregnancy. To date, many studies have investigated the role of miRNAs in regulating placental development, including trophoblast invasion, using multiple *in vitro*, *ex vivo* and *in vivo* models. Although several studies have suggested that some miRNAs play a role in the differentiation of enEVTs and SAR, our understanding of how miRNAs are involved in trophoblasts-mediated remodeling of uterine spiral arteries is still very limited.

Studies of miRNAs in the human placenta have been mostly carried out *in vitro.* While some papers reported the use of primary trophoblasts, placental villous explants, and villous explants/decidua co-culture, most published work have been done using established trophoblast cell lines. The use of a single cell line has its limitations considering the differences in marker gene expression and origin of many trophoblast cell lines (Pastuschek et al., 2021). Furthermore, the same cell line (e.g. HTR8/SVneo) in different labs appears to have different characteristics (Brkić et al., 2020). Cell line cross-contamination has also been reported (Nelson-Rees et al., 1981; Capes-Davis et al., 2010; Kniss and Summerfield, 2014; Ye et al., 2015). Additionally, some miRNAs might target different genes in different trophoblast cell lines (Chaiwangyen et al., 2015). Finally, while the use of animal models has advanced our understanding of miRNA functions in healthy and PE pregnancy (Rossant and Cross, 2001; Khalil and Granger, 2002; Carter, 2007; Palei et al., 2013; Rana et al., 2019; Frazier et al., 2020; Gatford et al., 2020; Bakrania et al., 2022), there are challenges in translating the findings to human applications. This is, in part, due to differences in placental structures between humans and non-primate models (Chaouat and Clark, 2015; Schmidt et al., 2015), the need for genetic, surgical or pharmacological manipulations to induce PE-like symptoms in these models (Kumasawa, 2018; Bakrania et al., 2022), and the fact that some miRNAs are not expressed in certain animals (Glazov et al., 2008; Noguer-Dance et al., 2010; Nayak et al., 2018; Schmidt et al., 2018). Therefore, it is important to use multiple systems such as cell lines, primary cells, *ex vivo* and *in vivo* models, as well as clinical samples, to study miRNA functions in human placentation and their involvement in the development of pregnancy-related disorders.

Many studies have reported miRNA expression profiles throughout gestation and their dysregulation in preeclamptic placentas and maternal serum; however, inconsistent findings are also often reported. This could be due to differences in the gestational age of the tissues, the composition of cell types in the samples, and clinical characteristics of the patients (Morales-Prieto et al., 2012; Pique-Regi et al., 2019; Smith et al., 2021). Thus, differential expression of miRNAs in healthy and PE placentas requires higher spatial resolution. Advanced techniques, such as single-cell sequencing, are powerful tools that will allow us to investigate miRNA expression and gene networks regulated by miRNAs within a cell population at unprecedented resolution. They will also reveal key sets of molecular events occurring throughout trophoblast differentiation, help elucidate molecular mechanisms underlying PE pathogenesis, and identify early PE markers to provide the means for early prediction and intervention of PE to improve maternal and fetal outcomes.
